# Early Maternal Deprivation Enhances Voluntary Alcohol Intake Induced by Exposure to Stressful Events Later in Life

**DOI:** 10.1155/2015/342761

**Published:** 2015-03-02

**Authors:** Sara Peñasco, Virginia Mela, Jose Antonio López-Moreno, María-Paz Viveros, Eva M. Marco

**Affiliations:** ^1^Departamento de Fisiología (Fisiología Animal II), Facultad de Ciencias Biológicas, Universidad Complutense de Madrid, Instituto de Investigación Sanitaria del Hospital Clínico San Carlos (IdISSC), 28040 Madrid, Spain; ^2^Departamento de Psicobiología, Facultad de Psicología, Universidad Complutense, 28223 Madrid, Spain

## Abstract

In the present study, we aimed to assess the impact of early life stress, in the form of early maternal deprivation (MD, 24 h on postnatal day, pnd, 9), on voluntary alcohol intake in adolescent male and female *Wistar* rats. During adolescence, from pnd 28 to pnd 50, voluntary ethanol intake (20%, v/v) was investigated using the two-bottle free choice paradigm. To better understand the relationship between stress and alcohol consumption, voluntary alcohol intake was also evaluated following additional stressful events later in life, that is, a week of alcohol cessation and a week of alcohol cessation combined with exposure to restraint stress. Female animals consumed more alcohol than males only after a second episode of alcohol cessation combined with restraint stress. MD did not affect baseline voluntary alcohol intake but increased voluntary alcohol intake after stress exposure, indicating that MD may render animals more vulnerable to the effects of stress on alcohol intake. During adolescence, when animals had free access to alcohol, MD animals showed lower body weight gain but a higher growth rate than control animals. Moreover, the higher growth rate was accompanied by a decrease in food intake, suggesting an altered metabolic regulation in MD animals that may interact with alcohol intake.

## 1. Introduction

Epidemiological data indicate that adverse life events during the first few years of life can increase the risk for psychopathology, including drug addiction [1, 2]. In fact, early life stress has been proposed to predict alcohol drinking in adolescence and alcohol abuse and dependence at adulthood (see [3] for review). Alcohol dependence affects millions of people worldwide, and alcohol consumption at adolescence has reached alarming rates [4]. In rodents, the effects of early life stress can be studied in a controlled manner by removal of the dam during the first 2 weeks of life (for a review on the animals models of dam-litter interaction disruption, please consult [5–7]). The effects of early life stress on alcohol consumption currently available are inconsistent and highly dependent on environmental factors such as the species and strains used and the protocol used for the maternal separation protocol (see [8] as an example). In rats, repeated maternal separation during early postnatal life (180–360 min. a day during the first three weeks of life) has been reported to induce an increase in alcohol intake [9–12], although no changes in alcohol consumption have also been reported [13–15]. It is worth mentioning that the investigation of possible sex differences in this regard has been neglected. In the last years, we have focused on the investigation of the early maternal deprivation (MD) animal model, that is, 24 h of dam-litter separation on postnatal day (pnd) 9. Adolescent animals exposed to MD have been reported to exhibit a trend to increased impulsivity, depressive-like responses, and impairments in cognitive function [16–18]. At adolescence, an increase in striatal dopamine levels has also been reported as a consequence of MD, possibly reflecting an altered brain reward system [19]. Actually, MD seems to modify the response of animals to diverse drugs of abuse, including cannabinoids [7, 18, 20], 3,4-methylenedioxymetamphetamine (MDMA) [21], and cocaine [22].

There is evidence about a relationship between stress and alcohol consumption (see [23, 24] for review) although the nature of this relationship is far from being understood. At this point, it is worth mentioning that, in a previous study, alcohol consumption during adolescence rendered animals more susceptible to acute stressor-specific effects in terms of alcohol consumption [25]. Therefore, in the present study alcohol deprivation was employed as a potential stressful stimulus that may influence motivation to drink [26, 27], and alcohol deprivation was also combined with restraint stress since exposure to restraint stress has been reported to increase alcohol consumption in rats [14].

In the present study we aimed to analyze the effects of MD on adolescent alcohol consumption, as well as addressing to which extent a previous history of early life stress (MD) could modulate the consequences of subsequent stressful life events (alcohol deprivation and alcohol deprivation combined with the concomitant exposure to restraint stress) on voluntary alcohol intake. Given the amount of evidence supporting sex differences in the consequences of MD, both male and female animals were employed in the present study.

## 2. Material and Methods

Experiments were performed in agreement with the European Directive 2010/63/EU on the protection of animals used for scientific purposes and in compliance with the Spanish Royal Decree 1201/2005, October 21, 2005 (BOE n° 252), about protection of experimental animals. All efforts were made to minimize animal suffering and distress.

### 2.1. Animals

Experiments were carried out on the offspring of rats purchased from Harlan Laboratories (Rossdorf, Germany), which were mated (one male × two females for ten consecutive days) in our animal facilities approximately 2 weeks after their arrival. After mating, female rats were housed individually in standard Plexiglas cages (50 × 25 × 17.5 cm.) and the animals were monitored daily for parturition. On the day of birth, postnatal day (pnd) 0, the litters were culled and sex balanced to 8 pups per dam (4 males and 4 females). No cross-fostering was allowed. Litters were randomly assigned to each experimental group: control (Co) and maternal deprivation (MD). All pups were weaned at pnd 22 and housed in pairs of sibling rats separated by sex. All animals were maintained in our animal facilities at the Faculty of Biological Sciences in the Complutense University of Madrid (EX08-UCS) at a constant temperature (21 ± 1°C) and humidity (60 ± 10%) in a reverse 12-hour dark-light cycle (lights on at 20.00 h), with free access to food (commercial diet for rodents A04/A03; SAFE, Augy, France) and water.

### 2.2. Early Maternal Deprivation (MD)

Early MD was performed as previously described [15]. In brief, on pnd 9, litters were submitted to 24 hours of maternal deprivation; that is, dams were removed from their home cages at 09.00 h, pups were weighted and remained undisturbed in the same room until the next day (pnd 10, 09.00 h) when pups were weighted again, and dams were placed back in their corresponding home cages. Pups from the control group (Co) were not manipulated but for body weight control on pnd 9 and pnd 10. Body weight was registered every six days until weaning (on pnd 16 and pnd 22). A total of 10 litters were used in the present experiment (5 Co litters and 5 MD litters); however, due to a technical mistake, 2 cages from the MD group had to be excluded from the study, and thus the total number of animals used was 76 (20 Co males and 20 Co females and 18 MD males and 18 MD females).

### 2.3. Voluntary Alcohol Consumption: Two-Bottle Choice

**Figure 1:**
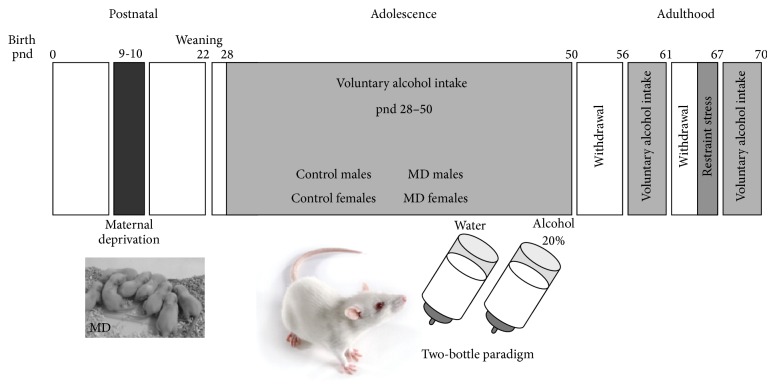
Schedule of experimental design. A total of ten litters were assigned to either of these two experimental groups: exposure to a single episode of early maternal deprivation (24 h on pnd 9, MD) or not deprived (control group, Co). All animals were given free access to alcohol (20%, v/v) and water in the two-bottle choice paradigm for (1) the whole adolescent period (from pnd 28 to pnd 50), for (2) four days (pnd 57 to pnd 60) after one week of alcohol cessation, and for (3) four days (pnd 67 to pnd 70) after a second period of alcohol cessation combined with 30 min. of restraint stress under white light conditions on the last three days of alcohol cessation. *n* = 18–20 animals* per *experimental group.

Once the rats became adolescents (pnd 28), rats were given free access to a bottle containing an ethanol solution (20% v/v) in addition to the water bottle. The ethanol solution (v/v) was prepared using 96° ethanol (Alcoholes Aroca S.L., Madrid, Spain) diluted in tap water. The positions of the bottles were changed daily to avoid position preference. Ethanol solution and water were changed every day. Measurements of alcohol and water intake were made daily during the recording periods, as well as body weight. Voluntary alcohol intake was recorded (1) during adolescence, from pnd 28 to pnd 50; (2) during a four-day period after one week of alcohol cessation, from pnd 57 to pnd 60; and (3) during a four-day period after a second period of alcohol cessation combined with exposure to restraint immobilization stress (30 min.* per* day under white light conditions) on the last three days of alcohol cessation, from pnd 67 to pnd 70. The amount of alcohol intake was expressed as grams (g.) and alcohol intake* per* body weight (kg.). Daily voluntary alcohol consumption was calculated during the three recording periods, and alcohol intake during the first 2 h. of alcohol exposure was also evaluated for the two last periods following exposure to additional stress (see experimental schedule for details, Figure 1). Body weight and food intake were controlled throughout the experimental period. During the evaluation of alcohol consumption animals remained paired housed to avoid possible interferences with the MD protocol due to isolation [28, 29].

### 2.4. Food Intake, Body Weight, and Growth Rate

Food intake was evaluated by placing a known amount of food in each cage and measuring the remaining amount after 24 hours. This procedure was performed during the adolescent period (voluntary alcohol consumption) and after the first and second alcohol cessation periods. Body weight was registered at pnd 9, pnd 16, and pnd 22 (before weaning) and daily thereafter during the periods of voluntary alcohol consumption, that is, adolescence, and following alcohol cessation. Body weight (BW) gain (in grams) was analyzed and calculated as the difference in BW referenced to pnd 9 during the preweaning period, pnd 28 during adolescence, and pnd 50 during the two periods of alcohol cessation. Additionally, growth rate (GR) was calculated as the percentage of BW gain divided by the reference BW employed during each period: GR (%) = (BW gain/BW_ref._) × 100.

### 2.5. Statistical Analyses

Data were analyzed using a two-way analysis of variance (ANOVA), considering sex (male or female) and neonatal condition (Co or MD) as independent factors. Shapiro-Wilk and Levene tests were used to confirm normality and homoscedasticity of the data. Repeated measures ANOVA was employed when appropriate, that is, BW gain and growth rate. Tukey post hoc comparisons were performed only for significant interactions, as well as additional one-way ANOVAs. Significance level was set at *P* < 0.05 for all comparisons. Statistical analyses were performed by the SPSS 19.0 software package (SPSS Inc., Chicago, IL, USA).

## 3. Results

### 3.1. Voluntary Alcohol Intake

#### 3.1.1. Voluntary Alcohol Intake during Adolescence

**Figure 2:**
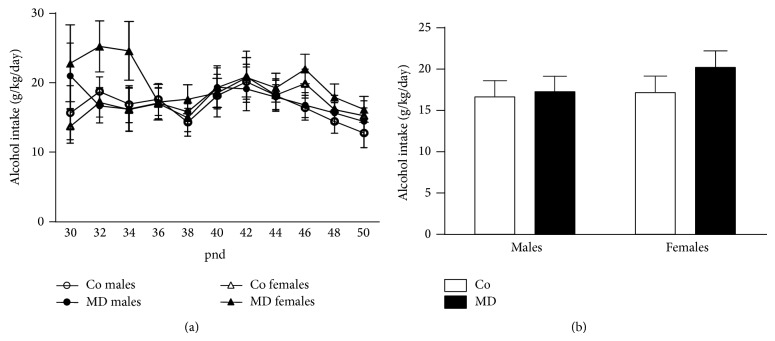
Voluntary alcohol intake during adolescence. (a) Daily alcohol intake (g/kg/day) along the whole adolescent period. Data are presented as mean ± S.E.M and were analyzed by a repeated measures two-way ANOVA. (b) Adolescent averaged alcohol intake (g/kg/day). Data were analyzed by a two-way ANOVA. Rats were exposed to a single episode of early maternal deprivation (24 h on pnd 9, MD) or not deprived (control group, Co) and exposed during adolescence, from postnatal day (pnd) 28 to pnd 50, to voluntary alcohol intake (20% v/v, in a two-bottle choice paradigm). *n* = 9-10 cages per experimental group.

No significant differences in alcohol intake were found during voluntary alcohol consumption at adolescence (control males: 16.6 ± 1.9 g/kg/day; control females: 17.2 ± 1.9 g/kg/day, MD males: 17.2 ± 2.0 g/kg/day; and MD females 20.2 ± 2.0 g/kg/day). Neither the repeated measures ANOVA nor the ANOVA analysis of the averaged alcohol intake during adolescence revealed any significant difference (see Figure 2). However, it is worth mentioning that MD females showed a trend for increased levels of alcohol intake during the first days of alcohol exposure.

#### 3.1.2. Voluntary Alcohol Intake following One Week of Alcohol Cessation

**Figure 3:**
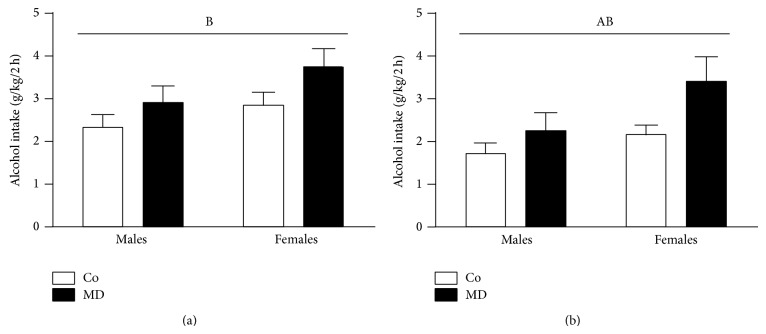
Voluntary alcohol intake following one week of alcohol cessation (a) and following a second week of alcohol cessation combined with restraint stress (b). Data are presented as mean ± S.E.M of the averaged alcohol intake (g/kg/2 h) during the first 2 h of alcohol exposure during the four days period of alcohol exposure (from pnd 57 to pnd 60, panel (a), and from pnd 67 to pnd 70, panel (b)). Rats were exposed to a single episode of early maternal deprivation (24 h on pnd 9, MD) or not deprived (control group, Co) and exposed to voluntary alcohol intake (20% v/v, in a two-bottle choice paradigm) during adolescence and following one week of alcohol cessation and after a second period of alcohol cessation combined with exposure to restraint immobilization stress (30 min.* per* day under white light conditions) on the last three days of alcohol cessation. Data were analyzed by a two-way ANOVA, *P* < 0.05; (A) general effect of sex, (B) general effect of the neonatal condition. *n* = 9-10 cages per experimental group.

Following one-week cessation, no significant differences were found on daily voluntary alcohol consumption (data not shown). However, a significant effect of the neonatal condition was found during the first 2 h of alcohol exposure (*F*(1,34) = 4.48; *P* < 0.05). Both male and female animals consumed more alcohol than their corresponding Co animals during the first 2 h of alcohol exposure (Figure 3(a)). Although not significant, a trend for higher levels of alcohol consumption was found for female animals when compared to males during these first 2 h of alcohol exposure (*F*(1,34) = 3.79; *P* = 0.06). No significant interaction between sex and neonatal condition was found.

#### 3.1.3. Voluntary Alcohol Intake following a Second Week of Alcohol Cessation Combined with Restraint Stress

Following a second period of alcohol cessation combined with restraint stress during the last three days, no significant differences were found on daily voluntary alcohol consumption (data not shown). However, a significant effect of the neonatal condition was found during the first 2 h of alcohol exposure (*F*(1,34) = 5.47; *P* < 0.05). MD animals exhibited a higher alcohol intake than Co animals during the period of alcohol exposure (Figure 3(b)). During this time period, a significant effect of sex appeared (*F*(1,34) = 5.30; *P* < 0.05). As for the previous time period, females showed higher levels of alcohol consumption compared to males during the first 2 h of alcohol exposure. No significant interaction between sex and neonatal condition was found.

### 3.2. Body Weight Gain and Growth Rate 

#### 3.2.1. Body Weight Gain and Growth Rate before Weaning

During the preweaning period, a significant effect of the neonatal condition was found when analyzing BW gain (*F*(1,16) = 89.88; *P* < 0.001) and growth rate (*F*(1,16) = 20.58; *P* < 0.001). Neither a significant effect of the sex condition nor a significant interaction between factors was found. The MD episode induced a significant reduction in BW gain and growth rate during the preweaning period (Table 1).

#### 3.2.2. Body Weight Gain and Food Intake during Adolescence

**Figure 4:**
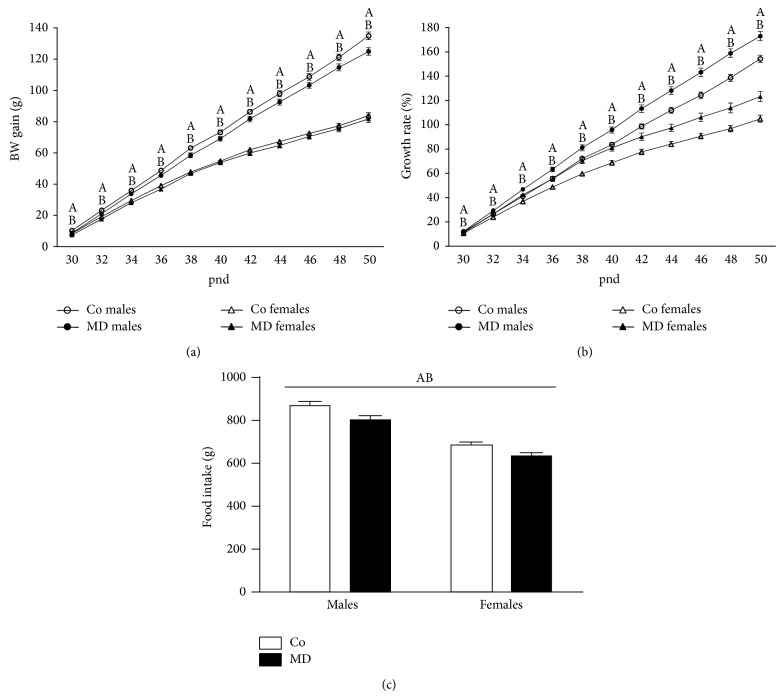
Metabolic parameters during adolescence in response to voluntary alcohol intake. (a) Body weight (BW) gain (g), (b) growth rate (%), and (c) accumulated food intake (g). Rats were exposed to a single episode of early maternal deprivation (24 h on pnd 9, MD) or not deprived (control group, Co) and exposed during adolescence, from postnatal day (pnd) 28 to pnd 50, to voluntary alcohol intake (20% v/v, in a two-bottle choice paradigm). Data are presented as mean ± S.E.M and were analyzed by a repeated measures two-way ANOVA (BW gain and growth rate) or by a two-way ANOVA (accumulated food intake). *P* < 0.05; (A) general effect of sex and (B) general effect of the neonatal condition; *n* = 18–20 animals* per *experimental group.

During adolescence, when animals had free access to alcohol, BW gain (Figure 4(a)) and growth rate (Figure 4(b)) were significantly affected by sex (*F*(1,72) = 281.62; *P* < 0.001; *F*(1,72) = 97.26; *P* < 0.001, resp.) and neonatal condition (*F*(1,72) = 6.47; *P* < 0.05; *F*(1,72) = 28.88; *P* < 0.001, resp.). No significant interaction between sex and neonatal condition was found. As expected, female animals showed lower BW gain and growth rate than male animals, while MD animals showed a lower BW gain but a higher growth rate than control animals. The analysis of accumulated food intake rendered a significant effect of sex (*F*(1,34) = 104.78; *P* < 0.001) with females eating less than males and a significant effect of the neonatal condition (*F*(1,34) = 11.57; *P* < 0.005) with MD animals consuming less food than their corresponding controls (Figure 4(c)). No significant interaction was found in this case.

#### 3.2.3. Body Weight (BW) Gain and Food Intake following One Week Alcohol Cessation and a Second Week of Alcohol Cessation Combined with Restraint Stress

**Figure 5:**
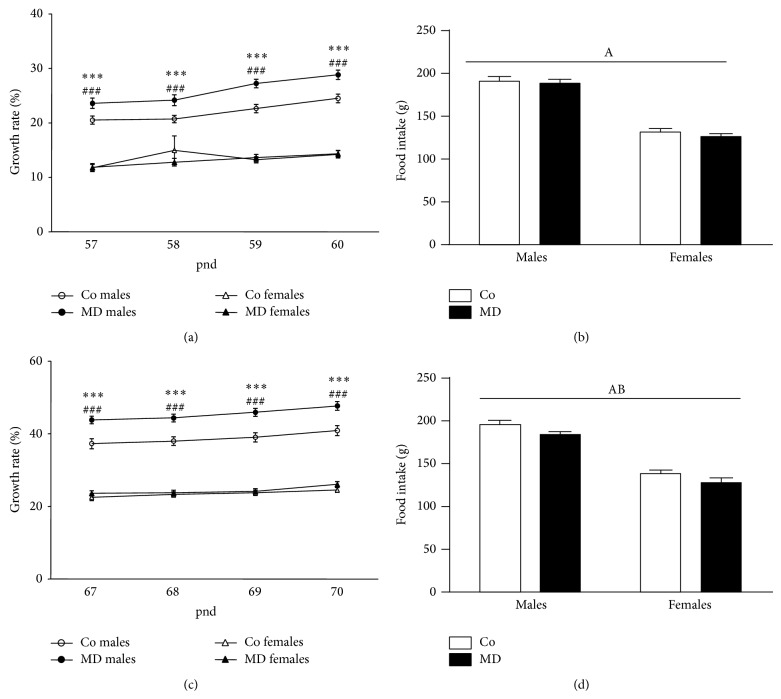
Metabolic parameters following stressful events in young adult rats in response to voluntary alcohol intake. ((a), (c)) Growth rate (%) and ((b), (d)) accumulated food intake (g). Rats were exposed to a single episode of early maternal deprivation (24 h on pnd 9, MD) or not deprived (control group, Co) and exposed to voluntary alcohol intake (20% v/v, in a two-bottle choice paradigm) during adolescence and following one week of alcohol cessation (from pnd 57 to pnd 60, upper panels) and after a second period of alcohol cessation combined with exposure to restraint immobilization stress (30 min.* per* day under white light conditions) on the last three days of alcohol cessation (from pnd 67 to pnd 70, lower panels). Data are presented as mean ± S.E.M and were analyzed by a repeated measures two-way ANOVA (BW gain). Tukey post hoc comparisons: ^***^ *P* < 0.005, MD males versus Co males; ^###^ *P* < 0.005, Co females versus Co males; or by a two-way ANOVA (accumulated food intake). *P* < 0.05; (A) general effect of sex, (B) general effect of the neonatal condition; *n* = 18–20 animals* per *experimental group.

A significant effect of sex on BW gain was found after the first (*F*(1,72) = 501.87, *P* < 0.001) and second (*F*(1,72) = 683.69; *P* < 0.001) periods of alcohol cessation, with females showing a lower BW gain than males across periods (data not shown). Similarly, the analysis of growth rate (Figures 5(a) and 5(c)) revealed a significant effect of sex after the first (*F*(1,72) = 242.03, *P* < 0.001) and second (*F*(1,72) = 341.55; *P* < 0.001) periods of alcohol cessation. Moreover, the analysis of growth rate also revealed a significant effect of the neonatal condition after the two alcohol cessation periods (first period: *F*(1,72) = 7.30; *P* < 0.01 and second period: *F*(1,72) = 14.65; *P* < 0.001). For the growth rate analyses, a significant interaction between sex and neonatal condition was also found (first period: *F*(1,72) = 8.14; *P* < 0.01 and second period: *F*(1,72) = 8.62; *P* < 0.005). During the two periods of alcohol exposure, female animals showed a lower growth rate than male animals, but MD only increased growth rate among male animals (no differences were observed among females). Regarding the analysis of the accumulated food intake (Figures 5(b) and 5(d)), a significant effect of sex was revealed (first period: *F*(1,34) = 210.18; *P* < 0.001 and second period: *F*(1,34) = 162.40; *P* < 0.001); males consumed more food than females during these two time periods. A significant effect of the neonatal condition was also found after the second period of alcohol cessation (*F*(1,34) = 6.29; *P* < 0.05); during this period MD animals consumed less food than control animals. No significant interaction between sex and neonatal condition was found for any of the time periods analyzed.

## 4. Discussion

Early life stress, in the form of a single 24-hour episode of MD at pnd 9, did not affect baseline voluntary alcohol intake during adolescence in rats of both sexes. However, MD seemed to exacerbate the effects of subsequent stress exposure on alcohol intake. Animals exposed to neonatal MD, compared to control non-MD animals, showed higher levels of alcohol intake following a short period of alcohol cessation as well as after the combination of an additional period of alcohol cessation concomitant to restraint stress exposure.

Under the present experimental conditions, MD,* per se*, did not appear to predict prospective alcohol intake during adolescence in either sex. Previous studies have investigated the impact of early life stress on voluntary alcohol intake, but different protocols of maternal separation were employed, that is, repeated and shorter episodes of maternal separation [8], and it is well known that different experimental procedures of this model of early life stress render different results. Moreover, most of the previous studies have investigated the effects of early life stress on adult animals, leaving adolescents out of the focus. Following repeated brief episodes of neonatal maternal separation, some studies reported an increase in alcohol intake [9–12], although other studies found no changes in alcohol consumption [13–15]. Discrepancies with previous reports may result from the timeframe selected for alcohol exposure, that is, adolescence; the protocol of early life stress employed, that is, MD; and differences in the protocol of alcohol exposure, that is, previous alcohol habituation, alcohol concentration, and/or choice between two or more bottles. However, present findings suggest MD animals to be more vulnerable to the acute effects of stress in terms of later alcohol consumption. In fact, MD animals showed higher levels of alcohol intake than control animals following a short period of alcohol cessation, and an additional short period of alcohol cessation combined with restraint stress. A previous study reported an increase in voluntary alcohol intake in adolescent animals exposed to a protocol of repeated maternal separation by using a paradigm of intermittent free access to alcohol. Since the paradigm of alcohol exposure employed in that study included brief periods of alcohol withdrawal [30], also considered as stressful stimuli (see below), those results could be considered similar to our present findings. Alcohol withdrawal has been considered as a potent stressor capable of disrupting several stress circuitries including the HPA axis [24] and has been proposed as a suitable model to study the impact of alcohol reexposure on relapse [31]. Moreover, a previous study that investigated the vulnerability of animals to the consequences of stress on alcohol intake revealed that alcohol deprivations in combination with more severe stress protocols, that is, electric foot shock, were able to increase alcohol consumption if animals had initiated alcohol intake during adolescence [25]. Similarly, Roman et al. [14] reported an increase in alcohol intake during the exposure in adult life to a restraint protocol and throughout the postrestraint period in male and female animals exposed to repeated periods of brief maternal separation. The relationship between stress and alcohol consumption is complex and poorly understood (see [23, 24] for review); however, present data suggest that early life stress and in particular MD may render animals more vulnerable to the consequences of future stressful situations regarding alcohol intake.

Early life stress has been associated with alterations in the development and establishment of several neurotransmitter circuitries involved in the stress response. As an example, MD has been reported to alter the development of the dopaminergic, serotonergic, and glutamatergic systems, known to be involved in alcohol effects and vulnerability [19, 32, 33]. A disruption of the endogenous cannabinoid system has also been reported as a consequence of MD [17, 34–38]. The endocannabinoid (eCB) system appears to be of special interest in the context of the present study since it has been proposed as one of the underlying mechanisms of alcohol use and abuse [39] and, more recently, as a putative candidate for the proclivity to alcohol intake observed in animals submitted to certain protocols of maternal separation [40]. Therefore, the changes induced by the MD episode on the eCB system may possibly underlie the increased alcohol intake reported after the exposure to stressful challenges later in life.

In humans, epidemiological studies indicate higher rates of alcohol abuse and dependence in men compared to woman [41]. However, data from animal models show the opposite; female animals seem more prone to consume alcohol than males [42, 43]. Discrepancies between human and animal studies may result from sociocultural factors, but the investigation of the underlying neurobiological substrates may help us to better understand these sex differences. In the present study, female animals consumed more alcohol than males in a two-bottle choice paradigm following an episode of alcohol cessation combined with restraint stress. Since sex differences in the response to stressors have been extensively described [44, 45], a differential response to stress in male and female animals might underlie, at least in part, the greater influence of stress on alcohol intake in females.

As in previous studies from our group, MD animals showed a significantly diminished BW gain until weaning when compared to Co animals [36, 46, 47], as well as a significantly decreased growth rate. However, these growing profiles changed after weaning. During adolescence, when animals had free access to alcohol, MD animals still showed lower BW gain than control animals, but they also showed a higher growth rate than controls. These results suggest that MD animals gain less body weight but they did it faster than control animals. The higher growth rate was accompanied by a decrease in food intake, suggesting an altered body weight regulation in MD animals that is evidenced in adolescence, coincident with alcohol exposure. It is worth mentioning that later in life, as evaluated following the exposure to stressful events, that is, alcohol cessation and alcohol cessation combined with restraint stress, only among male animals did the effects of MD on growth rate persist. Though a specific effect of weaning on the different growth rate of control and MD animals cannot be ruled out, it is important to point out that MD modifies the metabolic response to a metabolic challenge such as a high fat diet [48]. From this point of view, the present data may indicate that MD alters the metabolic response to alcohol, apparently in a sex dependent manner, though a direct confirmation of this hypothesis would require further experiments. We have previously reported that MD rats show a long lasting leptin deficiency (observed in neonates and adult rats) as well as an altered hypothalamic development [47, 49, 50]. Recently, de Timary et al. [51] have proposed a model for disruption of energy balance in alcohol dependent subjects in which altered leptin levels play a central role. The interactions between metabolic alterations induced by early life stress and those induced by alcohol abuse deserve further investigation, since adaptations of energy balance may contribute (aside from the effects of alcohol on the brain reward system) to the modulatory effects of MD on stress-induced alcohol intake.

## 5. Conclusions

Early maternal deprivation did not affect baseline voluntary alcohol consumption during adolescence but enhanced the levels of voluntary alcohol intake following exposure to stressful events later in life. Moreover, this episode of early life stress seemed to alter energy balance and metabolism since somatic growth and food intake were persistently modified in these animals. Future research is needed in order to clarify the impact of metabolic changes, such as altered leptin levels, on the susceptibility to increase alcohol intake in response to stress.

## Figures and Tables

**Table 1:** Metabolic parameters during the preweaning period.

	Males				Females			
	Control		MD		Control		MD	
	BW gain	Growth rate	BW gain	Growth rate	BW gain	Growth rate	BW gain	Growth rate
pnd 10	1.9 ± 0.1	9.0 ± 0.8	−0.8 ± 0.1^B^	−4.3 ± 0. 3^B^	1.7 ± 0.2	8.4 ± 1.2	−0.9 ± 0.1^B^	−4.6 ± 0.4^B^
pnd 16	15.0 ± 0.3	70.3 ± 4.2	9.0 ± 0.6^B^	46.5 ± 4.8^B^	13.6 ± 1.4	66.5 ± 8.0	8.8 ± 0.7^B^	46.8 ± 5.7^B^
pnd 22	37.3 ± 0.9	175.0 ± 9.7	27.9 ± 1.1^B^	143.0 ± 5.4^B^	34.0 ± 1.6	165.3 ± 10.4	26.7 ± 0.8^B^	140.5 ± 8.3^B^

Body weight gain (g) and growth rate (%) on different days during the preweaning period. Reference day was considered postnatal day, pnd, 9. Rats were exposed to a single episode of early maternal deprivation (24 h on pnd 9, MD) or not deprived (control group, Co). Data are expressed as mean ± S.E.M and were analyzed by a repeated measures two-way ANOVA, *P* < 0.05. ^B^General effect of the neonatal condition. *n* = 5 litters *per* experimental group.
